# Use of a 3D respiratory navigated IR-FLASH sequence after gadofosveset in the pediatric and adult congenital population

**DOI:** 10.1186/1532-429X-16-S1-P109

**Published:** 2014-01-16

**Authors:** Kevin K Whitehead, Matthew A Harris, Gary R McNeal, Mark A Fogel

**Affiliations:** 1 Cardiology and Radiology, Children's Hospital of Philadelphia, Philadelphia, Pennsylvania, USA; 2 CMR R&D, Siemens Healthcare, Chicago, Illinois, USA

## Background

While CMR has become instrumental in imaging complex congential heart disease (CHD), limitations remain including significant flow and metal artifact on cine images. 2D dark blood imaging generally requires gating to diastole which may not capture the maximal vessel dimensions, is time consuming and dependent on precise plane positioning. Traditional 3D whole heart sequences are also sensitive to flow and metal artifact. 3D respiratory navigated FLASH with an inversion recovery pulse (IR-FLASH) post-gadofosveset potentially overcomes all of these limitations. Furthermore, it is ideal for generating high resolution isotropic datasets of the blood pool for 3D modeling. We present our experience with IR-FLASH for imaging native and repaired CHD.

## Methods

We retrospectively reviewed our experience with IR-FLASH after gadofosveset. Respiratory navigating was employed with typical settings including isotropic voxels of 1.0 to 1.3 mm, TE 1.6 msec, TI 260 msec, and flip angle of 18 degrees. The TI was lowered to as low as 140 for systolic gating. Late systolic or diastolic quiescence was chosen for cardiac gating. We compared the IR-FLASH sequence to dynamic MRA, cine, and SSFP whole heart imaging when available.

## Results

IR-FLASH after gadofosveset was performed in 48 exams on 47 pts, with a mean age of 10.8 years (range 3 days to 32 years). Gating was performed to both diastole and systole in 22 cases, only systole in 4 cases, and only diastole in 22 cases. Image quality was judged to be good to excellent in 41 cases, and poor in only 2 cases. Systolic gating was generally successful for TI's of 180 or greater. Diagnoses included tetralogy of Fallot in 6 patients, unicuspid or bicuspid aortic valve in 5 cases, truncus arteriosus after repair in 4 cases, heterotaxy in 4 patients, and single ventricle status post cavopulmonary connections in 7 patients. In all patients with truncus or aortic valve disease, systolic gating precisely defined the valve morphology (see Image 2). Two patients had atrial septal stents that were well visualized, with only local artifact. Findings delineated that were missed by other modalities included pulmonary vein atresia in repaired total anomalous veins, a residual VSD in repaired DORV, and a thrombus in the native aorta of a pt with hypoplastic left heart and aortic atresia (see Image 1). Cavopulmonary pathways were well defined in all single ventricle pts. Coronary origins could be identified in 45 of the 48 IR-FLASH sequences.

**Figure 1:**
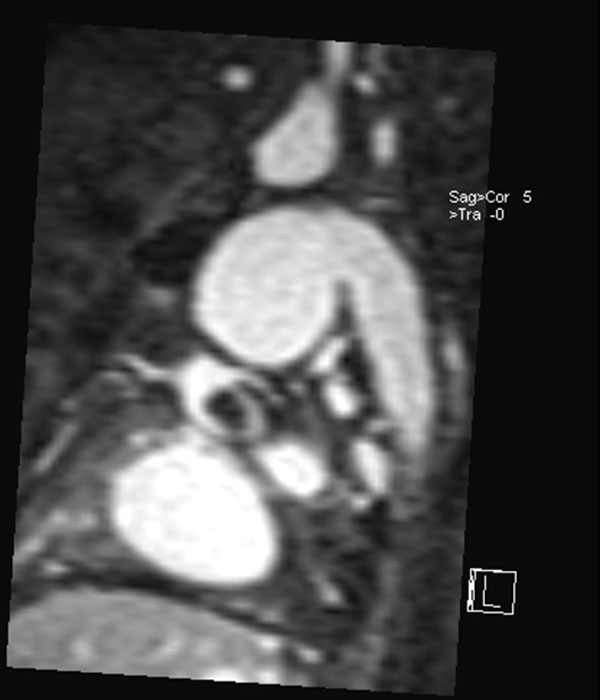
**Thrombus in native ascending aorta of patient with hypoplastic left heart syndrome after Fontan completion showing relationship to coronaries**.

**Figure 2:**
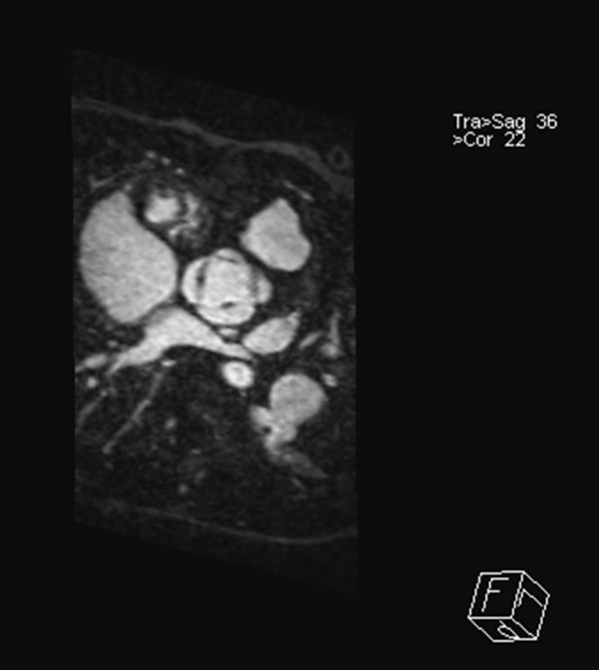
**Systolic gating in patient with repaired truncus arteriosus, delineating the quadricuspid truncal valve**.

## Conclusions

IR-FLASH after gadofosveset can be routinely implemented in a wide range of heart disease. It is less sensitive to flow and metal artifact than SSFP sequences. It can be reliably gated to both systole and diastole. Gating to systole allows for better definition of semilunar valve morphology, RVOT morphology, and maximal root dimensions. The high resolution, contrast, and ability to scan with isotropic voxels makes it ideal for generating 3D models for computational analysis or printing.

## Funding

None.

